# Supplementary material for: Neurobehavioral Alterations from Noise Exposure in Animals: A Systematic Review
Source: Int J Environ Res Public Health. 2022 Dec 29;20(1):591. doi: 10.3390/ijerph20010591 (PMC9819367; doi:10.3390/ijerph20010591)
Supplement: Supplementary file 1 [file ijerph-20-00591-s001.zip › ijerph-2031383-supplementary.pdf]

**Table S1.** Strings used for the online search.

| | |
|---|---|
| PUBMED (469) | ("noise*" OR "sound level" OR "noise pollution") AND ("exposure" OR "environment*") AND ("neurobehavioral" OR "behavioral" OR "psychological" OR "mental" OR "neurological") AND (animal*) NOT (human) |
| SCOPUS (712) | TITLE-ABS-KEY ( "noise" OR "sound level" OR "noise pollution" ) AND TITLE-ABS-KEY ( "exposure" OR "environment*" ) AND TITLE-ABS-KEY ( "neurobehavioral" OR "behavioral" OR "psychological" OR "mental" OR "neurological" ) AND TITLE-ABS-KEY ( animal ) AND NOT TITLE-ABS-KEY ( human ) |
| EMBASE (81) | ('animal'/exp OR'animalia' OR 'metazoa' OR 'animal' OR 'animal population groups' OR 'animals' OR 'metazoan' OR 'metazoans' OR 'metazoon') AND ('noise'/exp OR 'electric noise' OR 'furnace noise' OR 'noise' OR 'noise break' OR 'noise burst signal' OR 'noise intensity' OR 'noise level' OR 'noise sound' OR 'noisy environment') AND ('pollution'/exp OR 'environment contamination' OR 'environment pollution' OR 'environmental pollution' OR 'pollution' OR 'pollution, environmental') AND ('behavior disorder'/exp OR 'aberrant behavior' OR 'aberrant behaviour' OR 'behavior aberration' OR 'behavior disorder' OR 'behavior disorder, child' OR 'behavior disturbance' OR 'behavioral crisis' OR 'behavioral disorder' OR 'behavioral disturbance' OR 'behaviour aberration' OR 'behaviour disorder' OR 'behaviour disorder, child' OR 'behaviour disturbance' OR 'behavioural crisis' OR 'behavioural disorder' OR 'behavioural disturbance' OR 'child behavior disorder' OR 'child behavior disorders' OR 'child behaviour disorder' OR 'child behaviour disorders' OR 'child reactive disorders' OR 'deviant behavior' OR 'deviant behaviour' OR 'disturbed behavior' OR 'disturbed behaviour' OR 'elopement' OR 'neurobehavioral manifestations') |
| WEB OF SCIENCE (360) | ((((ALL=(("noise*" OR "sound level" OR "noise pollution") )) AND ALL=(("exposure" OR "environment*") )) AND ALL=(("neurobehavioral" OR "behavioral" OR "psychological" OR "mental" OR "neurological") )) AND ALL=(animal*)) NOT ALL=(human*) |
| COCHRANE LIBRARY (9) | (("noise*" OR "sound level" OR "noise pollution")):ti,ab,kw AND (("exposure" OR "environment*")):ti,ab,kw AND (("neurobehavioral" OR "behavioral" OR "psychological" OR "mental" OR "neurological")):ti,ab,kw AND (animal*):ti,ab,kw |

ABS, ab = abstract; ti= title; kw= keywords.
